# Quality of age data in the Sierra Leone Ebola database

**DOI:** 10.11604/pamj.2020.35.104.20348

**Published:** 2020-04-07

**Authors:** Lindsay Shively Womack, Charles Alpren, Frederick Martineau, Amara Jambai, Tushar Singh, Reinhard Kaiser, John Terrell Redd

**Affiliations:** 1 Epidemic Intelligence Service, Center for Surveillance, Epidemiology and Laboratory Services, Centers for Disease Control and Prevention, Atlanta, Georgia, USA; 2 Division of Vital Statistics, National Center for Health Statistics, Centers for Disease Control and Prevention, Hyattsville, Maryland, USA; 3 United States Public Health Service, Rockville, Maryland, USA; 4 London School of Hygiene and Tropical Medicine, London, UK; 5 Ministry of Health and Sanitation, Freetown, Sierra Leone; 6 Sierra Leone Country Office, Division of Global Health Protection, Center for Global Health, Centers for Disease Control and Prevention, Freetown, Sierra Leone; 7 Center for Preparedness and Response, Centers for Disease Control and Prevention, Washington DC, USA

**Keywords:** Sierra Leone Ebola outbreak, data quality, terminal digit preference, age heaping

## Abstract

**Introduction:**

While it is suspected that some ages were misreported during the 2014-2016 West African Ebola outbreak, an analysis examining age data quality has not been conducted. The study objective was to examine age heaping and terminal digit preference as indicators for quality of age data collected in the Sierra Leone Ebola Database (SLED).

**Methods:**

Age data quality for adult patients was analyzed within SLED for the Viral Hemorrhagic Fever (VHF) database and the laboratory testing dataset by calculating Whipple´s index and Myers´s blended index, stratified by sex and region.

**Results:**

Age data quality was low in both the VHF database (Whipple´s index for the 5-year range, 229.2) and the laboratory testing dataset (Whipple´s index for the 5-year range, 236.4). Age was reported more accurately in the Western Area and least accurately in the Eastern Province. Age data for females were less accurate than for males.

**Conclusion:**

Age data quality was low in adult patients during the 2014-2016 Ebola outbreak in Sierra Leone, which may reduce its use as an identifying or stratifying variable. These findings inform future analyses using this database and describe a phenomenon that has relevance in data collection methods and analyses for future outbreaks in developing countries.

## Introduction

The West African Ebola outbreak in 2014-2016 resulted in over 28,000 cases and 11,000 deaths [1]. Deficiencies in pre-existing public health infrastructure information systems in Sierra Leone exacerbated data collection difficulties and complicated the public health response to the outbreak [2]. The Sierra Leone Ministry of Health and Sanitation (MoHS) used the Centers for Disease Control and Prevention (CDC) Epi Info Sierra Leone Viral Hemorrhagic Fever (VHF) application as a surveillance system to monitor the epidemic [3,4]. The resulting VHF database contains clinical information, such as symptoms and date of onset and demographic data reported by suspected case patients or their relatives and collected by case investigators [3]. While this database is often used for national and international level analyses because it provides the most comprehensive epidemiologic data on Ebola cases available in Sierra Leone, there were considerable difficulties encountered in ensuring consistency and completeness of the data [5]. These difficulties impaired contact tracing and links with other databases such as those created or recorded by the Ebola Treatment Centers´ data managers and the burial teams, which experienced similar data collection and quality problems. The Sierra Leone MoHS, with assistance from the CDC, consolidated available records to form a more comprehensive and complete database, referred to as the Sierra Leone Ebola Database (SLED) [6].

At the time of the epidemic, Sierra Leone did not have a widely used unique identifier system for the population (e.g. equivalent to a social security number in the United States). Careful recording of personal information, such as name and age of the patient, was particularly important during the Ebola outbreak to ensure accurate connection of laboratory testing results to individual patients, avoid duplication within a database and ensure accurate comparisons across databases. However, it is common for individuals in Sierra Leone to be unaware of their exact age and not to possess a birth certificate, even if their birth was registered with the Registrar of Births and Deaths [7].

The tendency of reporting certain ages instead of others (e.g. rounding to the nearest age that ends in '0' or '5'), referred to as age heaping, has been shown previously in Sierra Leone census data and survey data [8-10]. In addition to complicating the linkage of databases, inaccurately reported age data can have implications for the quality of analyses using the data. While it is suspected that there was a preference of reporting ages with a terminal digit of either '0' or '5' during the Ebola virus disease outbreak, an analysis to examine the quality of age data from the SLED database has not been conducted.

This study examined the quality of age data collected for adult patients during the Ebola virus disease outbreak. Our objective was to describe age heaping as an indicator for inaccurate age data collected during the Ebola virus disease outbreak in Sierra Leone, with the goal of informing future SLED analyses and assessing implications for data management of other large-scale public health responses. The project was approved by the Sierra Leone MoHS and the CDC IRB.

## Methods

**Data:** within the SLED database, data were analyzed separately for the VHF database and the laboratory testing dataset for the years 2014 and 2015. A de-identified analytic project data package was prepared by SLED data managers in Sierra Leone and transferred to the National Center for Health Statistics Research Data Center (RDC) [11] by secure file transfer protocol for analysis. Records with missing values for age or sex were excluded (3.0% from the VHF database and 7.3% from the laboratory testing dataset). To maintain confidentiality, analysis groups by sex and region with less than 15 records were suppressed. In addition, an RDC analyst reviewed the output to prevent personal-identifiable information (PII) disclosure. This study also served as a testing project for providing secure data access to SLED.

**VHF study dataset:** records for adult patients with recorded age were included in the VHF study dataset. Records for children patients were not included because the ages of childhood are less affected by age heaping. SLED data managers in Sierra Leone extracted two datasets for analysis: a national dataset by single age and a dataset by age group and region, to avoid PII disclosure. The exact method of age documentation for each individual was not analyzed. For instance, there is no indication in the datasets if a patient provided their own age data or if another individual gave the information.

**Laboratory testing study dataset:** records for initial laboratory tests for adult patients were included in the laboratory testing study dataset. Records for children patients were not included because the ages of childhood are less affected by age heaping. Because laboratory testing results were recorded for each sample tested, we only included initial tests (generally the first test for the patient) to exclude follow-up testing records for the same patient. SLED data managers in Sierra Leone extracted the data from the laboratory testing dataset by age group to avoid small counts. The exact method of age documentation for each patient was not recorded.

### Measurement of age heaping and age accuracy

**VHF study dataset:** age distribution was analyzed using a single-year age plot by sex (national data only). Age heaping was calculated using Whipple´s index [12], stratified by sex and region of residence (Western Area, Northern Province, Eastern Province, Southern Province). Terminal digit preference was calculated using Myers´s blended index [12], stratified by sex (national data only).

**Laboratory testing study dataset:** age heaping was calculated using Whipple´s index, stratified by sex and region of residence. Additionally, a sub-analysis was conducted on patients who were tested for the Ebola virus prior to their death, for the Western Area and Northern Province. This sub-analysis was conducted to determine if age was collected more or less accurately in patients who were tested for Ebola virus prior to death. Terminal digit preference could not be calculated because SLED data managers in Sierra Leone extracted the data by age group to avoid small counts.

**Whipple´s index:** it was developed to detect a preference or avoidance for ages ending in '0', '5', or both [12]. The index measures age heaping in the range of 23 to 63 years and assumes uniform distribution within a 5 or 10 year range. The ages of childhood (<20 years) and old age (>79 years) are excluded because they are more strongly affected by other types of error of reporting than by terminal digit preference [12]. The formula to calculate Whipple´s index is as follows:

Whipple´s index for the 10-year range:

$$=\frac{\sum \left(P_{30}+P_{40}+P_{50}+P_{60}\right)}{\frac{1}{10}\sum \left(P_{23}+P_{24}+P_{25}+\cdot \cdot \cdot +P_{60}+P_{61}+P_{62}\right)}\times 100$$

Whipple´s index for the 5-year range:

$$=\frac{\sum \left(P_{25}+P_{30}+\cdot \cdot \cdot +P_{55}+P_{60}\right)}{\frac{1}{5}\sum \left(P_{23}+P_{24}+P_{25}+\cdot \cdot \cdot +P_{60}+P_{61}+P_{62}\right)}\times 100$$

P represents that size of the population for each single-year age group. Whipple´s index varies between 100 and 500, with 100 indicating no preference for ages ending in '0' or '5' and 500 indicating only ages ending in '0' and '5' were reported [12]. The United Nations scale for interpreting Whipple´s index is as follows: <105 = highly accurate; 105-109.9 = fairly accurate; 110-124.9 = approximate; 125-174.9 = rough; ≥175 = very rough [13]. The proportion of individuals with an age reported with a terminal digit of '0' and '5' was evaluated using a two-tailed z-test for difference of proportion at the 0.05 level.

**Myers´s blended index:** Myers´s blended index is similar to Whipple´s index, except that it detects the preference or avoidance for ages ending in any of the ten digits [12]. The index assumes that the population is equally distributed among the different ages. Therefore, the expected frequency of all ten digits is ten percent. Myers´s blended index indicates the preference for each terminal digit, represented as a deviation from ten percent. A summary index of preference for all terminal digits was also calculated. The theoretical range of Myers´s blended index is between 0 and 90, with 0 representing no heaping and 90 occurring if all ages were reported with the same terminal digit. Myers´s blended index was calculated for the range of 20 to 79 years because the index is less affected by extreme of ages [12].

## Results

**Figure 1:**
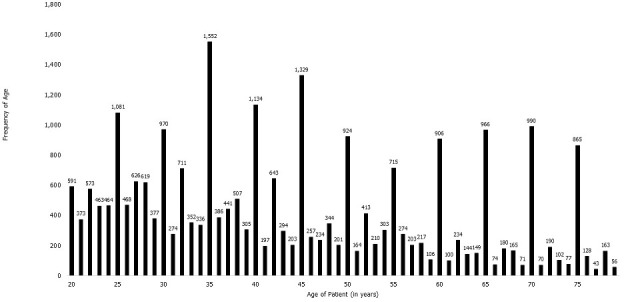
Single-year age distribution of males who had Ebola virus test outcome data in the Viral Hemorrhagic Fever (VHF) dataset: Sierra Leone, 2014-2015

**Figure 2:**
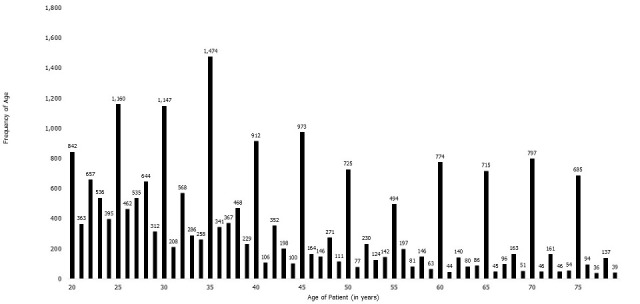
Single-year age distribution of females who had Ebola virus test outcome data in the Viral Hemorrhagic Fever (VHF) dataset: Sierra Leone, 2014-2015

**VHF dataset:** the age data of 46,660 individuals between the ages of 20 and 79 years were included in the study population from the national VHF dataset. The single-age national distribution for males and females are shown in Figure 1 and Figure 2, respectively. Both men and women show heaping for ages ending in both '0' and '5'.

The total number of individuals in the regional VHF dataset between the ages of 23 and 62 was 35,497. Among them, 7,492 (21.1%) individuals had ages reported ending with '0' and 8,778 (24.7%) individuals had ages reported ending with '5'. Whipple´s index for the 10-year range (i.e. ages ending in '0') was 211.1 overall, 201.4 for males, and 222.9 for females (Table 1). Whipple´s index for the 5-year range (i.e. ages ending in '0' or '5') was 229.2 overall, and 220.4 for males and 239.9 for females (Table 1).

**Table 1:** Whipple’s index for individuals who had Ebola virus test outcome data in the Viral Hemorrhagic Fever (VHF) dataset: Sierra Leone, 2014-2015

	Both Sexes	Males	Females	p-value*
**Whipple’s index for the 10-year age range (number of individuals)**				
Total	211.1 (35,497)	201.4 (19,537)	222.9 (15,960)	<0.001
Western Area	165.5 (15,381)^†^	164.2 (8,741)^†^	167.2 (6,640)^†^	0.62
Northern Province	238.5 (11,937)	227.1 (6,095)	250.4 (5,842)	<0.01
Eastern Province	270.5 (5,117)^§^	252.9 (3,009)^§^	295.5 (2,108)	<0.001
Southern Province	238.9 (2,842)	215.6 (1,563)	267.4 (1,279)	<0.01
**Whipple’s index for the 5-year age range (number of individuals)**				
Total	229.2 (35,497)	220.4 (19,537)	239.9 (15,960)	<0.001
Western Area	182.7 (15,381)^†^	178.2 (8,741)^†^	188.6 (6,640)^†^	<0.01
Northern Province	265.3 (11,937)	256.2 (6,095)	274.8 (5,842)	<0.001
Eastern Province	275.0 (5,117)^§^	264.9 (3,009)	289.4 (2,108)^§^	<0.001
Southern Province	250.9 (2,842)	235.8 (1,563)	269.4 (1,279)	<0.001

* p-value for differences between males and females

† Significantly lower Whipple’s Index than all other regions (p < 0.05)

§ Significantly higher Whipple’s Index than all other regions (p < 0.05)

NOTE: Total number of individuals does not equal sum of region due to individuals with unknown region

The Western Area had the most individuals included in the study population (8,741 males and 6,640 females), followed by the Northern Province (6,095 males and 5,842 females). Whipple´s index for the 10-year range varied by region, ranging from 165.5 in the Western Area to 270.5 in the Eastern Province (Table 1). Whipple´s index for the 5-year range followed a similar pattern, ranging from 182.7 in the Western Area to 275.0 in the Eastern Province (Table 1). Females had a higher Whipple´s index for both the 10-year and 5-year range than males for all regions, except the 10-year range in the Western Area (Table 1).

**Figure 3:**
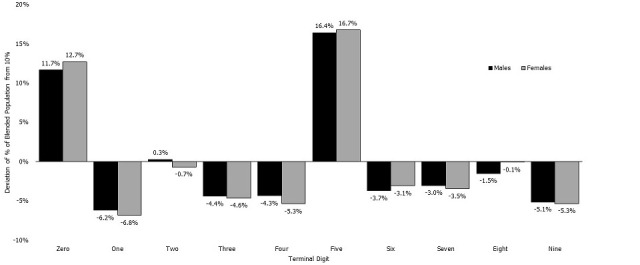
Terminal digit preference in the age of individuals who had Ebola virus test outcome data in the Viral Hemorrhagic Fever (VHF) dataset by sex: Sierra Leone, 2014-2015

The Myers´s blended index for the national study population was 29.65 overall, 28.34 for males, and 29.43 for females. Preference or avoidance for ages ending in any of the ten digits by sex is shown in Figure 3. Preference for ages ending in '0' and '5' was shown for both men and women.

**Laboratory testing study dataset:** the total number of individuals between the ages of 23 and 62 included in the study population from the laboratory testing dataset was 18,698 (Table 2). Among them, 4,029 (21.6%) individuals had ages reported ending with '0' and 4,812 (25.7%) individuals had ages reported ending with '5'. Whipple´s index for the 10-year range was 215.5 overall, 209.0 for males and 223.5 for females (Table 2). Whipple´s index for the 5-year range was 236.4 overall, and 229.0 for males and 245.6 for females (Table 2).

**Table 2:** Whipple’s index for individuals in the laboratory testing dataset: Sierra Leone, 2014-2015

	Both Sexes	Males	Females	p-value
**Whipple’s index for the 10-year age range**				
Total	215.5 (18,698)	209.0 (10,350)	223.5 (8,348)	0.02
Western area	152.6 (7,404)^†^	151.6 (4,216)^†^	154.0 (3,188)^†^	0.77
Northern province	245.2 (6,410)	232.6 (3,392)	259.4 (3,018)	0.01
Eastern province	274.4 (2,405)	269.1 (1,390)	281.8 (1,015)	0.49
Southern province	269.1 (2,479)	267.0 (1,352)	271.5 (1,127)	0.80
**Whipple’s index for the 5-year age range**				
Total	236.4 (18,698)	229.0 (10,350)	245.6 (8,348)	<0.001
Western area	175.9 (7,404)^†^	169.8 (4,216)^†^	184.0 (3,188)^†^	0.01
Northern province	271.5 (6,410)	261.2 (3,392)	283.0 (3,018)	<0.001
Eastern province	291.3 (2,405)^§^	287.4 (1,390)	296.6 (1,015)	0.37
Southern province	273.3 (2,479)	272.9 (1,352)	273.7 (1,127)	0.94

*p-value for differences between males and females

† Significantly lower Whipple’s Index than all other regions (p < 0.05)

§ Significantly higher Whipple’s Index than all other regions (p < 0.05)

The study population from the laboratory testing dataset ranged from 2,405 individuals (1,390 males and 1,015 females) in the Eastern Province to 7,404 individuals (4,216 males and 3,188 females) in the Western Area. Whipple´s index for the 10-year range ranged from 152.6 overall (151.6 males; 154.0 females) in the Western Area to 274.4 overall (269.1 males; 281.8 females) in the Eastern Province (Table 2). Whipple´s index for the 5-year range followed the same pattern, with the lowest index in the Western Area (175.9 overall; 169.8 males; 184.0 females) and the highest index in the Eastern Province (291.3 overall; 287.4 males; 296.6 females) (Table 2). Females had a higher Whipple´s index for both the 10 year and 5 year range than males overall and in the Northern Province. Females had a higher Whipple´s index for the 5-year range in the Western Area.

Within the laboratory testing dataset, the Whipple´s index for individuals with an initial blood test result for the Ebola virus prior to their death was lower than all individuals within the dataset for the Western Area and Northern Province (Table 3). Age distribution and terminal digit preference could not be assessed due to small counts in order to maintain the confidentiality of individuals.

**Table 3:** Whipple’s index for individuals with an initial blood test result for the Ebola virus prior to death compared with all individuals in the laboratory testing dataset: Sierra Leone, 2014-2015

	Initial blood test prior to death	All individuals	p-value*
**Whipple’s index for the 10-year age range**			
Western area	144.9 (4,457)	152.6 (7,404)	0.26
Northern province	202.3 (2,754)	245.2 (6,410)	<0.001
**Whipple’s index for the 5-year age range**			
Western province	171.0 (4,457)	175.9 (7,404)	0.27
Northern province	229.1 (2,754)	271.5 (6,410)	<0.001

* p-value for differences between individuals with an initial blood test results for the Ebola virus prior to death and all individuals in the laboratory testing dataset

## Discussion

Our analysis revealed significant age heaping in two essential databases from the 2014-2016 Ebola outbreak in Sierra Leone. Preference for ages ending in '0' and '5' was present in both men and women. The Eastern, Northern and Southern provinces in Sierra Leone had a Whipple´s index greater than 175, indicating very rough age distribution with a preference of reporting ages with terminal digits '0' and '5'. Only the Western Area of Sierra Leone had a Whipple´s index values between 125 and 175, indicating a rough age distribution. Myers´s blended index for the VHF dataset indicates that a minimum of 29% of the patients had reported ages with an incorrect final digit.

Inaccurate single age data in SLED Ebola outbreak data can lead to misclassification bias and inaccurate assessments of age-specific Ebola rates. Furthermore, our results confirm that single age was of limited use as an identifying variable in Sierra Leone during the Ebola outbreak. While this is the first analysis of the quality of age data collected during the 2014-2016 West African outbreak, substantial evidence of misreporting of age has been documented for the Sierra Leone Demographic and Health Survey, both in 2008 and in 2013 [8,10].

Terminal digit preference is not limited to populations in Sierra Leone nor to age data alone. Inaccurately reporting age is common in demographic studies and has also been shown in clinical cohorts [12,14]. In demographic studies, preference for ages ending with terminal digits of '0' and '5' was correlated with low education level [12]. Digit preference bias has also been previously described in situations when patients are asked to report data such as year of menopause, smoking rate and in situations when clinicians are responsible for recording measurements such as blood pressure and birthweight [15-18]. Emergency departments also show considerable digit preference bias in the recording of patient time of departure from the emergency department [19,20].

There are several possible explanations related to the Ebola outbreak for the inaccurate age data reported in SLED. First, both the reporter and the recorder influence what number is entered for age. As mentioned earlier, substantial evidence of misreporting of age has been documented for population-based samples in Sierra Leone previously [8,10], indicating that the misreporting of age in the SLED Ebola outbreak data may be a reflection of the Sierra Leone national behavior. However, because we cannot determine what method exactly was used to document age, we do not know if it was the patient themselves reporting or a family member or neighbor who reported the age (e.g. if the patient was too ill to self-report or if the patient had died prior to reporting). Depending on the identity and relationship of that family member or neighbor, it is possible that they did not know the patient´s exact age. Alternatively, the person recording or collecting the data may have estimated the patient's age in cases where the patient or a proxy was unavailable to respond. Both types of estimates may have favored reporting ages ending in either '0' or '5'. Data quality in Sierra Leone may have been further exacerbated by the crisis situation [21], which may have implications for data collection and reporting of data in future humanitarian emergencies.

In 2011, the United Nations office for the Coordination of Humanitarian Affairs issued a report stating that information gaps on sex and age limits the effectiveness of humanitarian response in all phases of a crisis [22]. The report argues that proper collection, analysis and use of sex and age disaggregated data allows operational agencies to deliver assistance more effectively and efficiently [22]. The SLED Ebola outbreak data collection and maintenance effort is commendable in that age data were collected to better inform the outbreak response and to inform analyses of the outbreak. However, our study results highlight the difficulties of collecting accurate age data to be used as an identifying or stratifying variable during humanitarian emergencies, especially in developing countries where age may already be more likely to be misreported [14]. In Sierra Leone, efforts are underway to improve civil registration, which may result in better availability and knowledge of birth dates and exact ages [23].

Our study has limitations. We were unable to determine if age was reported by the patient or by proxy. While this indication would not alter the accuracy of the age data, it would allow us to determine where the source of error might have originated. Additionally, we were not able to calculate Myers´s blended index in the laboratory testing dataset or by region in the VHF dataset. This analysis would have allowed us to detect the preference or avoidance for ages ending in any of the ten digits and not only ages ending in '0' or '5'. However, our overall conclusion that the quality of age data was poor would remain the same. Finally, best efforts were made to de-duplicate records in both the VHF dataset and laboratory testing dataset; however, a small number of duplicate records may have remained in the files.

This study highlights that during humanitarian emergencies, age data may be collected inaccurately. Specifically, our study shows that age data quality was low during the 2014-2016 Ebola outbreak in Sierra Leone, and therefore may have had limited use as an identifying or stratifying variable. In addition to informing future analyses using this database, these findings describe a phenomenon that may have relevance in data collection methods for future humanitarian emergencies.

## Conclusion

Age data quality was low in adult patients during the 2014-2016 Ebola outbreak in Sierra Leone, which may reduce its use as an identifying or stratifying variable. These findings inform future analyses using this database and describe a phenomenon that has relevance in data collection methods and analyses for future outbreaks in developing countries.

### What is known about this topic

- Deficiencies in pre-existing public health infrastructure information systems in Sierra Leone exacerbated data collection difficulties and complicated the public health response to the West African Ebola outbreak in 2014-2016;
- The tendency of reporting certain ages instead of others (e.g. rounding to the nearest age that ends in '0' or '5'), referred to as age heaping, has been shown previously in Sierra Leone census data and survey data.

### What this study adds

- Our analysis revealed significant age heaping in two essential databases from the 2014-2016 Ebola outbreak in Sierra Leone;
- Our study shows that age data quality was low during the 2014-2016 Ebola outbreak in Sierra Leone, and therefore may have had limited use as an identifying or stratifying variable;
- These findings describe a phenomenon that may have relevance in data collection methods for future humanitarian emergencies, in addition to informing future analyses using this database.

## Competing interests

The authors declare no competing interests.

## References

[1] World Health Organization Ebola Situation Reports 2016.

[2] World Health Organization (2015). Factors that contributed to undetected spread of the Ebola virus and impeded rapid containment.

[3] Dietz PM, Jambai A, Paweska JT, Yoti Z, Ksiazek TG (2015). Epidemiology and risk factors for Ebola virus disease in Sierra Leone-23 May 2014 to 31 January 2015. Clin Infect Dis.

[4] CodePlex Archive The Epi Info Viral Hemorrhagic Fever Application 2019.

[5] McNamara LA, Schafer IJ, Nolen LD, Gorina Y, Redd JT, Lo T (2016). Ebola surveillance - Guinea, Liberia and Sierra Leone. MMWR Suppl.

[6] CDC (2019). Sierra Leone Ebola Database (SLED).

[7] Statistics Sierra Leone - SSL, ICF International (2014). Sierra Leone Demographic and Health Survey 2013.

[8] Lyons-Amos M, Stones T (2017). Trends in demographic and health survey data quality: an analysis of age heaping over time in 34 countries in sub-Saharan Africa between 1987 and 2015. BMC Res Notes.

[9] Bailey M, Makannah TJ (1996). An evaluation of age and sex data of the population censuses of Sierra Leone: 1963-1985. Genus.

[10] Pullum T, Staveteig S (2017). An assessment of the quality and consistency of age and date reporting in DHS surveys, 2000-2015.

[11] National Center for Health Statistics NCHS Research Data Center (RDC) 2018.

[12] Swanson D, Siegel J (2004). The methods and materials of demography.

[13] United Nations (2018). United Nations Demographic Yearbook 2016.

[14] Denic S, Khatib F, Saadi H (2004). Quality of age data in patients from developing countries. J Public Health (Oxf).

[15] Crawford SL, Johannes CB, Stellato RK (2002). Assessment of digit preference in self-reported year at menopause: choice of an appropriate reference distribution. Am J Epidemiol.

[16] Klesges RC, Debon M, Ray JW (1995). Are self-reports of smoking rate biased, evidence from the second national health and nutrition examination survey. J Clin Epidemiol.

[17] McManus RJ, Mant J, Hull MR, Hobbs FD (2003). Does changing from mercury to electronic blood pressure measurement influence recorded blood pressure, an observational study. Br J Gen Pract.

[18] Edouard L, Senthilselvan A (1997). Observer error and birthweight: digit preference in recording. Public Health.

[19] Keep SL, Locker TE (2012). The impact of a computerised whiteboard system on digit preference bias in the recording of emergency department process times. Eur J Emerg Med.

[20] Locker TE, Mason SM (2006). Digit preference bias in the recording of emergency department times. Eur J Emerg Med.

[21] Boland ST, Polich E, Connolly A, Hoar A, Sesay T, Tran AA (2017). Overcoming Operational Challenges to Ebola Case Investigation in Sierra Leone. Glob Health Sci Pract.

[22] Mazurana Dyan, Benelli Prisca, Gupta Huma, Walker Peter (2011). Sex and age matter: improving humanitarian response in emergencies.

[23] National Civil Registration Authority National Civil Registration Authority 2018.

